# Noble Gases Therapy in Cardiocerebrovascular Diseases: The Novel Stars?

**DOI:** 10.3389/fcvm.2022.802783

**Published:** 2022-03-16

**Authors:** Jiongshan Zhang, Wei Liu, Mingmin Bi, Jinwen Xu, Hongzhi Yang, Yaxing Zhang

**Affiliations:** ^1^Department of Traditional Chinese Medicine, The Third Affiliated Hospital, Sun Yat-sen University, Guangzhou, China; ^2^Institute of Integrated Traditional Chinese and Western Medicine, Sun Yat-sen University, Guangzhou, China; ^3^Department of Physiology, School of Basic Medical Sciences, Guangzhou University of Chinese Medicine, Guangzhou, China; ^4^Research Centre for Integrative Medicine (Key Laboratory of Chinese Medicine Pathogenesis and Therapy Research), Guangzhou University of Chinese Medicine, Guangzhou, China; ^5^Department of Otorhinolaryngology, The Seventh Affiliated Hospital, Sun Yat-sen University, Shenzhen, China

**Keywords:** helium (He), neon (Ne), argon (Ar), krypton (Kr), xenon (Xe), cardiovascular diseases, cerebrovascular disease, gasoimmunology

## Abstract

Cardiocerebrovascular diseases (CCVDs) are the leading cause of death worldwide; therefore, to deeply explore the pathogenesis of CCVDs and to find the cheap and efficient strategies to prevent and treat CCVDs, these are of great clinical and social significance. The discovery of nitric oxide (NO), as one of the endothelium-derived relaxing factors and its successful utilization in clinical practice for CCVDs, provides new ideas for us to develop drugs for CCVDs: “gas medicine” or “medical gases.” The endogenous gas molecules such as carbon monoxide (CO), hydrogen sulfide (H_2_S), sulfur dioxide (SO_2_), methane (CH_4_), and hydrogen (H_2_) have essential biological effects on modulating cardiocerebrovascular homeostasis and CCVDs. Moreover, it has been shown that noble gas atoms such as helium (He), neon (Ne), argon (Ar), krypton (Kr), and xenon (Xe) display strong cytoprotective effects and therefore, act as the exogenous pharmacologic preventive and therapeutic agents for CCVDs. Mechanistically, besides the competitive inhibition of N-methyl-D-aspartate (NMDA) receptor in nervous system by xenon, the key and common mechanisms of noble gases are involved in modulation of cell death and inflammatory or immune signals. Moreover, gases interaction and reduction in oxidative stress are emerging as the novel biological mechanisms of noble gases. Therefore, to investigate the precise actions of noble gases on redox signals, gases interaction, different cell death forms, and the emerging field of gasoimmunology, which focus on the effects of gas atoms/molecules on innate immune signaling or immune cells under both the homeostatic and perturbed conditions, these will help us to uncover the mystery of noble gases in modulating CCVDs.

## Introduction

Cardiovascular diseases (CVDs) are a group of disorders of heart and blood vessels, CVDs include primary hypertension, pulmonary arterial hypertension, abdominal aortic aneurysm, coronary heart disease (CHD) (especially myocardial ischemia, which is primarily mediated by the buildup of atherosclerotic plaque in the blood vessels that supply oxygen and nutrients to the heart, coronary artery vasospasm, and coronary microvascular dysfunction) (1–4), congenital heart disease, valvular heart disease (e.g., rheumatic heart disease), myocarditis and inflammatory cardiomyopathy, diabetic cardiomyopathy, and other conditions, ultimately cardiac arrhythmias and/or heart failure; additionally, cerebrovascular diseases (CBVDs), a range of conditions influencing brain and cerebral arteries, e.g., ischemic stroke, also belong to CVDs; therefore, CVDs also refer to as cardiocerebrovascular diseases (CCVDs) (5–13). Heart attack and stroke are the representative diseases of CCVDs (14). CCVDs are the leading cause of death globally and the statistical data from the WHO indicate that CCVDs take an estimated 17.9 million lives each year (15). Therefore, to deeply explore the pathogenesis of CCVDs and to find the cheap and efficient strategies to prevent/treat CCVDs, these are of great clinical and social significance.

The discovery of nitric oxide (NO), as one of the endothelium-derived relaxing factors (for which the Nobel Prize in Physiology or Medicine was awarded in 1998) and its successful clinical application in CCVDs, opens a new direction for the scientists to discover drugs for treating CCVDs: “medical gases” or “gas medicine” (16–18). For example, the endogenous gases, including carbon monoxide (CO), hydrogen sulfide (H_2_S), sulfur dioxide (SO_2_), methane (CH_4_), and hydrogen (H_2_, which is primarily produced by intestinal flora), have been shown to prevent or treat CCVDs in animals or in human body (19–32). Recently, noble gas family has emerged as the novel exogenous pharmacologic preventive and therapeutic agents for CCVDs (33–38). The aim of this comprehensive review is to summarize and discuss the current understanding of the biological effects and mechanisms of noble gases on CCVDs.

## Basic Characteristics of Noble Gases

**Figure 1:**
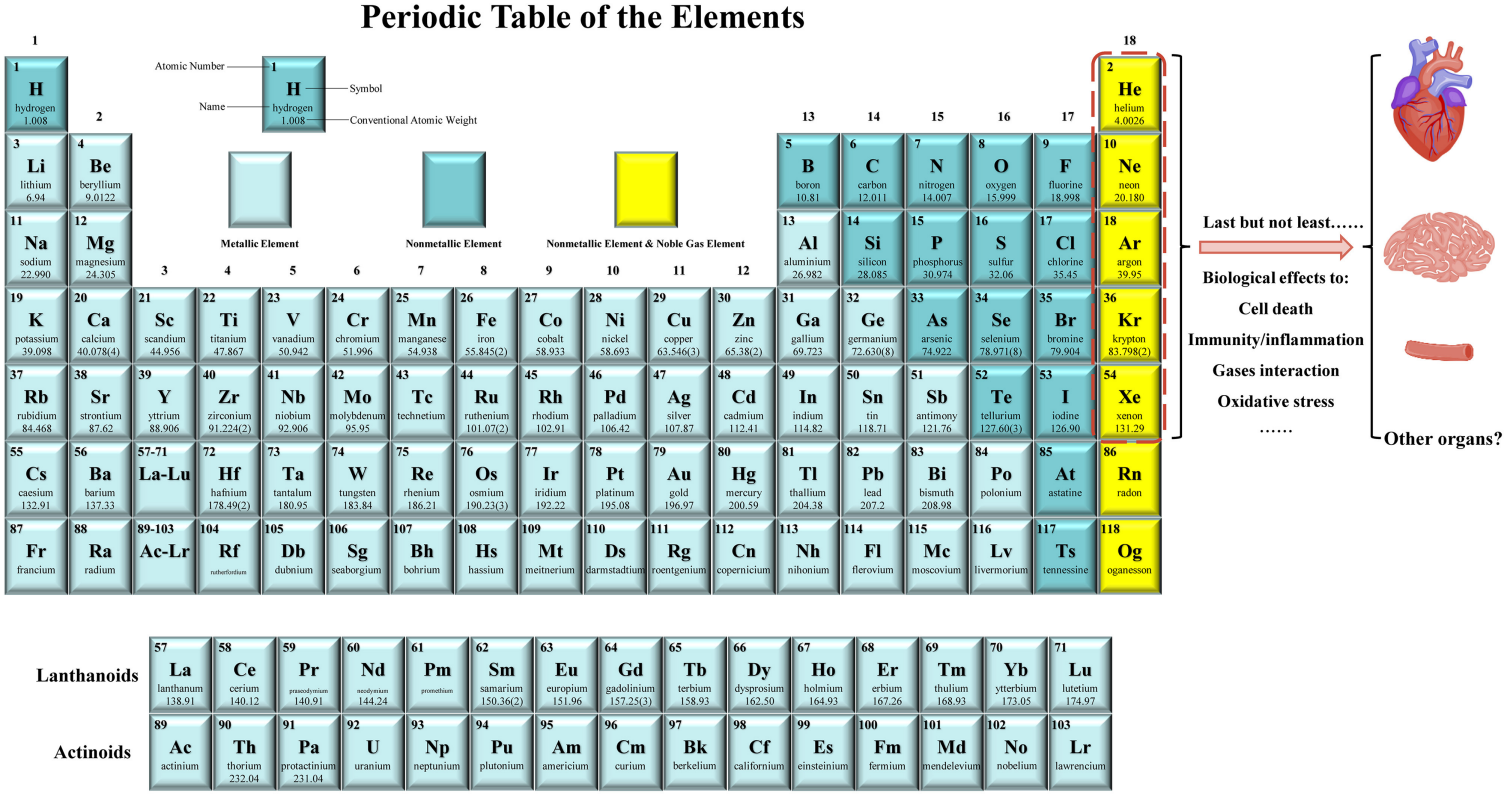
Noble gases have emerged as the novel preventive and therapeutic agents for cardiocerebrovascular diseases (CCVDs). The noble gas family includes helium (He), neon (Ne), argon (Ar), krypton (Kr), xenon (Xe), and the radioactive radon (Rn). They are monatomic gases at the far right of the periodic table and are chemically inert. Last but not least, it has been shown that most of noble gases have essential biological effects, including modulation of cell death, immunity/inflammation, gases interaction, and oxidative stress. They have been acted as protectants for alleviating the injuries of heart, brain, blood vessels (e.g., endothelial cells), liver, kidney, and intestine in animal models or in human body. Therefore, noble gases therapy provides a novel idea for the prevention and treatment of CCVDs.

The noble gases refer to the gas atoms corresponding to all the group 18 elements on the periodic table of the elements. This family constitutes six naturally occurring gases: helium (He), neon (Ne), argon (Ar), krypton (Kr), xenon (Xe), and the radioactive radon (Rn) (38) (Figure 1). Xe was first shown to possess anesthetic properties in 1951, whereas none of the other five noble gases show anesthetic properties under normobaric conditions (38, 39). At normal temperature and normal pressure, noble gases are odorless, colorless, and monatomic gases that are characterized by a filled outer shell of valence electrons, making them “inert” or at least less capable of interaction with other compounds; therefore, they are also known as “inert gases” (34–36, 38). However, some of these noble gases have strong biological activities such as the properties of neuroprotection and cardioprotection (34–36, 38).

## Noble Gases: The “New World” of Cardiocerebrovascular Protection

### Helium

Helium is the second most abundant element in the universe after H_2_; however, He is only sixth element in the composition of dry air (0.00052%) (40). It is the lightest noble gas with an atomic weight of 4 g/mol and has the lowest melting (−458°F, −272.2°C) and boiling (−452.1°F, −268.9°C) points of all the elements (36, 40). Due to the lower density and viscosity, heliox-21 (21% oxygen and 79% helium), which weight is one-third compared with air, can reduce work of breathing; therefore, heliox has been reported to be effective in a variety of respiratory conditions, including asthma exacerbation, post-extubation stridor, croup, upper airway obstruction, bronchiolitis, acute respiratory distress syndrome (ARDS), chronic obstructive pulmonary disease (COPD), and pulmonary function testing (36, 40, 41). He has the lower solubility than nitrogen; the mixture of helium and oxygen rather than nitrogen and oxygen decreases the formation of nitrogen bubbles and, therefore, alleviating decompression illness in deep-sea divers (41). Moreover, He is safe for abdominal insufflation and may be the insufflating agent of choice in patients with significant cardiopulmonary disease and laparoscopic renal surgery (42–44). He inhalation enhanced vasodilator effect of inhaled NO on pulmonary vessels in hypoxic dogs; this enhanced vasodilatory effect of NO on He might be associated with facilitated diffusion of NO diluted in the gas mixture with He (45). In the past decade, a series of studies showed that He has essential cytoprotective effects on endothelial cells (ECs) (46–48), heart (49–60), brain (59, 61–67), liver (68), and intestine (69).

#### Helium in Endothelial Protection

Caveolin-1 (Cav-1) was secreted after He exposure *in vitro*, altered the cytoskeleton, and increased the adherent junction protein vascular endothelial-cadherin (VE-cadherin) and gap junction protein connexin 43 (Cx43) expression thus, resulting in decreased permeability in ECs (47). These indicated that He protected endothelium by maintaining barrier function and preventing leakage and tissue edema and ultimately preserving endothelial function (47). Furthermore, the plasma of healthy volunteers breathing He protected ECs against hypoxic cell damage by increasing Cav-1 expression and Cav-1 knockdown in ECs abolished this effect (48); the interesting question is what contents from the plasma contribute to these effects. However, another study showed that pretreating with He increased ECs damage *in vitro* under the stimulation of tumor necrosis factor-α (TNF-α) or hydrogen peroxide (H_2_O_2_) (70).

Helium can induce preconditioning in human endothelium *in vivo*: inhalation of 3 cycles of heliox21 for 5 min, followed by 5 min of normal air breathing either directly before forearm ischemia (20 min) or 24 h before forearm ischemia (20 min), attenuated ischemia-reperfusion (I/R)-induced endothelial dysfunction independent of endothelial NO synthase (eNOS), as that the protection of He was not abolished after blockade of eNOS (46). However, *Eliana Lucchinetti* et al. (71) showed that heliox-50 (50% helium and 50% oxygen), breathing from 15 min before ischemia until 5 min after the onset of reperfusion, provided modest anti-inflammatory effects, but did not restore endothelial dysfunction of the forearm in humans *in vivo*. A case report indicated that accidental inhalation of He under high pressure can cause symptomatic cerebral and coronary artery gas embolism (72). Therefore, the concentration, time, and mode (continuously or intermittently) of supplying He, the different pathological stimuli, and *in vivo* and *in vitro* might be responsible for the above controversies.

#### Helium in Cardioprotection

Helium preconditioning (HePC) can considerably reduce infarct size in myocardial I/R injury model of rabbits, young rats but not aged rats, Zucker lean rat but not Zucker obese rats (49–51, 73) (Table 1). These He-induced cardioprotection are related to activating phosphoinositide 3-kinase (PI3K), p44/42 mitogen-activated protein kinase (MAPK) (ERK1/2), p70S6 kinase (p70s6K), cyclic AMP (cAMP)-dependent protein kinase (PKA), cyclooxygenase-2 (COX-2), opioid receptors, mitochondrial Ca^2+^-sensitive potassium channel, and mitochondrial ATP-regulated potassium (KATP) channels (possibly producing small quantities of ROS); inhibiting mitochondrial permeability transition pore (mPTP) opening and NO production by eNOS (49, 50, 53, 73–76, 78). Moreover, suppression of glycogen synthase kinase-3 (GSK-3) or p53 lowered the threshold of He-induced preconditioning *via* the mPTP-dependent mechanism *in vivo* (77). He also induced post-conditioning in the myocardial I/R injury model of Zucker lean rats or male Wistar rats, these protective effects on Wistar rats are related to increasing genes involved in autophagy, inhibiting genes involved in apoptosis, increasing protein levels of Cav-1/3, and activating ERK1/2 and Akt (51, 56, 57) (Table 1). However, inhaled 30 or 60 min of 70% He during reperfusion dose does not induce cardioprotection in male adult Wistar rats (55). This process was not accompanied by reducing the hyperacute burst of inflammatory cytokines, but the prolonged He inhalation might contribute to the proinflammatory response, such as increasing cytokine-induced neutrophil chemoattractant 3 (CINC-3) and interleukin-1β (IL-1β) in myocardium from area at risk, but not from area not at risk (55). Moreover, one clinical investigation indicated that HePC (3 ×5 min of 70% He and 30% oxygen was applied before aortic cross-clamping), helium post-conditioning (15 min of He was applied before release of the aortic cross-clamp and was continued for 5 min after begin of reperfusion) or the combination had no effects on the activation of p38 MAPK, ERK1/2, or on the levels of protein kinase C-epsilon (PKC-ε) and heat-shock protein 27 (HSP27) in patient hearts undergoing coronary artery bypass graft surgery; HePC and helium post-conditioning did not affect postoperative troponin release in these patients (58). In contrast to the healthy Wistar Kyoto rats (WKRs), only a triple intervention of He conditioning can reduce cell damage after myocardial I/R in spontaneous hypertensive rats (SHR), suggesting the presence of a threshold in the hypertensive heart (54) (Table 1). An *in-vitro* study indicated that He conditioning contributed to cardioprotection by increasing fibroblast migration, but not by releasing protective medium extracellular vesicles or soluble factors from the cardiac fibroblasts (88). Our recent study indicated that intraperitoneal injection of 99.999% He gas improved lipopolysaccharide (LPS)-induced left ventricular dysfunction and cavity enlargement in a dose-dependent manner, it is better at the dose of 1.0 ml/100 g (89). Mechanistically, He inhibited Toll-like receptor 4 (TLR4) expression, reduced the phosphorylation of nuclear factor-kappa B (NF-κB), and subsequently alleviated interleukin-18 (IL-18) and TNF-α expression in heart (89). The dose effect of He gas has also been confirmed in intestine; the HePC profile consisting of three cycles of 10 or 15 min He breathing interspersed with three 5-min washout periods by breathing room air reduced I/R-induced intestinal injury, inflammatory response, and cell apoptosis; however, the 2- or 5-min He breathing dose does not confer any protective effects (69).

**Table 1:** Noble gases alleviate myocardial ischemia/reperfusion (I/R) injury in animal models.

**Noble gases**	**Animals**	**Doses and Time**	**Key Results**	**References**
Helium	Male New Zealand white rabbits (2.5–3.0 kg)	Rabbits received 1, 3 or 5 cycles of 70% He-30% O_2_ for 5 min interspersed with 5 min of 70% N_2_-30% O_2_ or an air-oxygen mixture before ischemia	Reduced infarct size	(49–51, 53, 54, 56, 57, 73–78)
	Young male Hannover Wistar rats (352 ± 15 g)	Rats received 70% He-30% O_2_ for three 5-min periods, interspersed with two 5-min washout periods 10 min before ischemia		
	Male Wistar rats (~328 g)	Rats received 70% He-30% O_2_, 50% He-30% O_2_-20% N_2_, or 30% He-30% O_2_-40% N_2_ for 15 min 24 h before ischemia, or received 30% He-30% O_2_-40% N_2_ for 15 min on 3, 2, or 1 day(s), interspersed by 24 h, respectively		
	Zucker lean rat (238–262 g)	Rats received 70% He-30% O_2_ for three 5-min periods, interspersed with two 5-min washout periods 10 min before ischemia, or inhaled 70% He-30% O_2_ for 15 min at the onset of reperfusion		
	Male Wistar rats (354–426 g)	Rats were subjected to 25 min ischemia and 15 min reperfusion, and 70% He-30% O_2_ post-conditioning (PostC) encompassed the entire reperfusion phase		
	Male Wistar Kyoto rats (WKR) and spontaneous hypertensive rats (SHR) (12–14 weeks)	PostC, Late preconditioning (LPC) + PostC, or Early preconditioning (EPC) + LPC + PostC was performed in WKR. EPC + LPC + PostC was performed in SHR. EPC comprised 3 short cycles of 70% He-30% O_2_ (5 min each, with wash outs of 5 min in between and a final washout episode of 10 min before ischemia). LPC was induced by 15 min of 70% He-30% O_2_ administration 24 h before ischemia. PostC was induced by 15 min of 70% He-30% O_2_ administration since the beginning of reperfusion		
Neon	Male New Zealand white rabbits (2.5–3.0 kg)	Rabbits received 3 cycles of 70% Ne-30% O_2_ for 5 min interspersed with 5 min of 70% N_2_-30% O_2_ before ischemia	Reduced infarct size	(73)
Argon	Male New Zealand white rabbits (2.5–3.0 kg)	Rabbits received 3 cycles of 70% Ar-30% O_2_ for 5 min interspersed with 5 min of 70% N_2_-30% O_2_ before ischemia	Reduced infarct size	(73, 79)
	Male Wistar rats (240–380 g)	Inhalation of 80% Ar-20% O_2_ for 20 min starting 5 min before reperfusion	Preserved left ventricular function at 1 and 3 weeks after surgery	
Krypton	No report yet	No report yet	No report yet	None
Xenon	New Zealand white rabbits (2.7–3.4 kg)	Inhalation of 70% Xe-30% O_2_ during first 15 min of reperfusion	Reduced infarct size	(80–87)
	Male Wistar rats (275–350 g)	Administration of 20% Xe-80% O_2_ was commenced 3 min prior to, and discontinued 30 min after, the onset of reperfusion. Moreover, active cooling was commenced 5 min prior to, and hypothermia maintained for 1 h after, the onset of reperfusion		
	Male Wistar rats (200–250 or 300–450 g)	Rats received 70% Xe-25% O_2_-5% N_2_ for three 5-min periods, interspersed with two 5 min and one final 10-min washout periods before ischemia		
	Male Wistar rats (280–340 g)	Rats received 3 cycles of 70% Xe-30% O_2_ administered for 5- min periods interspersed with 5 -min intervals 70% N_2_-30% O_2_ following by a final 15-min interval of 70% N_2_-30% O_2_ before ischemia		
	Male Wistar rats (200–250 g)	24 h before ischemia, rats received 70% Xe-30% O_2_ for 15 min		
Radon	No report yet	No report yet	No report yet	None

#### Helium in Neuroprotection

Helium displayed neuroprotective effects on a traumatic brain injury model *in vitro* (61) and in a decompression-induced neurological deficits model *in vivo* (90). Breathing 70% He during a middle cerebral artery occlusion (MCAO) for 2 h and early reperfusion (1 h) reduced infarct volume and improved neurological deficits 24 h after MCAO in rats (91). Seventy-five percentage He treatment from 1 h after reperfusion to 4 h after reperfusion also provided neuroprotection by producing hypothermia in rats (62). In a rat resuscitation model, HePC and He post-conditioning (received 70% He and 30% oxygen for 5 min before cardiac arrest and for 30 min after restoration of spontaneous circulation) reduced apoptosis in brain, but had no influence on viable neuron count and no beneficial effects were seen on neurofunctional outcome (59). He-PC-induced NO production and subsequent NO-mediated up-regulation of antioxidases (e.g, nuclear factor E2-related factor 2), angiogenesis, and inhibition of inflammation and apoptosis, all contributed to the neuroprotective effect of helium in a neonatal cerebral hypoxia/ischemia model (63, 65, 66). However, in a clinical perspective for the treatment of acute ischemic stroke, He should not be administered before or together with tissue plasminogen activator therapy due to the risk of inhibiting the benefit of tissue plasminogen activator-induced thrombolysis; He therapy could be an efficient neuroprotective agent, if given after tissue plasminogen activator-induced reperfusion (64).

#### Helium in Hepatic Protection

Fukuda et al. have confirmed that inhalation of H_2_ gas (1–4% at 10 min before reperfusion until the end of reperfusion) suppressed hepatic I/R (90/180 min) injury through reducing oxidative stress in male C57 BL/6N mice (4–5 weeks old, 15–18 g); however, 4% He gas showed no protective effect (92). Similarly, HePC (three cycles of ventilation with inhalation of mixture of 70% He and 30% oxygen for 5 min, each followed by 5-min washout with inhalation of mixture of 30% oxygen and 70% nitrogen) did not attenuate hepatic I/R (45/240 min) injury in male Wistar rats (300 ± 30 g), although there was evidence for a modulation of the inflammatory response (93). In contrast, Zhang et al. have revealed that HePC (70% He-30% oxygen mixture inhalation for three 5-min periods interspersed with three 5-min washout periods using room air) alleviated 90 min ischemia-induced liver injury at 1, 3, and 6 h after reperfusion in male BALB/c mice (25–30 g); mechanistically, activation of hepatic adenosine A_2A_ receptor-PI3K-Akt axis, alleviation of necrosis and apoptosis, reduction of IκBα phosphorylation, and TNF-α, interleukin-6 (IL-6), monocyte chemotactic protein-1 (MCP-1) and chemokine (C-X-C motif) ligand 10 (CXCL10, IP-10) expression, and inhibition of inflammatory cell infiltration in liver all contributed to this protective effects of HePC (68). The difference of animal strains, the time of I/R, and even the gas mixture used in washout periods might be responsible for these controversy. Furthermore, Zhang et al. have confirmed that HePC-induced protection in hepatic I/R injury and Akt activation were dependent on the interaction between He inhalation and air gaps, but not any of the two factors alone (68). As the protection of the intermittent pattern of He inhalation, drinking hydrogen-rich water, or intermittent hydrogen gas exposure, but not lactulose or continuous hydrogen gas exposure, prevented 6-hydroxydopamine-induced Parkinson's disease in rats (94). Therefore, the continuous heliox inhalation, rather than intermittent pattern, might be responsible for the none alteration of myocardial infarct size or the extent of no reflow in rabbits with continuous heliox breathing during 30 min of ischemia and 180 min of reperfusion (95).

### Argon

#### Argon in Neuroprotection

When 7-day-old postnatal Sprague-Dawley rats subjected to hypoxic-ischemia (moderate) injury, 2 h after hypoxic insult, exposure of He, Ar, and Xe (70% noble gas balanced with oxygen) for 90 min improved cell survival, brain structural integrity, and neurologic function on postnatal day 40 compared with nitrogen, whereas only Ar and Xe reduced infarct volume after more severe hypoxic-ischemic injury (96). The *in-vivo* and *in-vitro* studies indicated that Ar acted as a protector for cerebral ischemia injury, brain trauma, and cardiac arrest-induced neurological damage (97–110). The neuroprotective effects of Ar were involved in inhibiting microglia/macrophage activation and enhancing M2 microglia/macrophage polarization (107, 109, 110), reducing stress-activated protein kinase/c-Jun N-terminal kinase (SAPK/JNK) activation and high mobility group protein B1 (HMGB1) expression (106), inhibiting TLR2/4-mediated activation of signal transducer and activator of transcription 3 (STAT3) and NF-κB, and subsequently decreasing IL-8 expression (111).

#### Argon in Cardioprotection

Argon displayed cardioprotective effects both *in vitro* and *in vivo* (73, 79, 106, 112, 113). Pre-treatment with 30 or 50% Ar for 90 min before oxygen-glucose deprivation protected human cardiac myocyte-like progenitor cells against apoptosis *via* activation of ERK, Akt, and biphasic regulation of JNK (113). Preconditioning with three cycles of 50% Ar (50% Ar, 21% oxygen, and 29% nitrogen) for 5 min, interspersed with 5 min of 79% nitrogen-21% oxygen *in vivo*, enhanced post-ischemic cardiac functional recovery following cardioplegic arrest and global cold ischemia *in vitro*; this protective effect of Ar was related to improving cardiac energy metabolism, inhibiting JNK phosphorylation, and HMGB1 expression (106). The cardioprotection of Ar on ischemia was also confirmed in rabbit *in vivo* (73) (Table 1). Lemoine et al. have further revealed the therapeutic effect of Ar on left ventricular dysfunction in myocardial I/R injury *in vivo*, in which Ar activated PI3K/Akt mitogen-activated protein kinase kinase (MEK)-ERK1/2 signaling, inhibited the opening of mitochondrial permeability transition pore (79) (Table 1).

#### Argon in Hepatic Protection

Argon is the key modulator of IL-6 expression in different liver injury models. Under the physiological conditions, IL-6 is essential for proper hepatic tissue homeostasis, liver regeneration, infection defense, and fine-tuning of metabolic functions, while persistent activation of IL-6 seems to be detrimental, impairs liver regeneration and can even lead to the development of liver cancer (114, 115). Inhalation of 50% Ar inhibited liver regeneration after hepatic I/R or after partial hepatectomy in rats, the former may be related to upregulation of IL-1β and IL-6 in liver, and the latter may be related to the downregulation of hepatocyte growth factor (HGF) and IL-6 (116, 117). Breathing 70% Ar in a rabbit model of abdominal aorta occlusion for 30 min and reperfusion for 300 min also reduced the plasma concentrations of IL-6 and HMGB1, improved hepatic and renal injuries (118). The detail mechanisms of Ar-mediated IL-6 expression are not clear.

### Xenon

#### Xenon in Neuroprotection

As that of Ar, Xe also has essential neuroprotective effects and it has been extensively investigated in the animal models of ischemia- and/or hypoxia-induced nervous system damage, such as, stroke, brain trauma, and hypoxic-ischemic injury in rat hippocampus (61, 96, 119–135). Glutamate mediates most excitatory neurotransmission in the mammalian central nervous system; normal activation of glutamate receptors mediates, in large measure, physiological excitatory synaptic transmission in the brain and is, therefore, crucial for the normal functioning of nervous system (136, 137). However, among three classical glutamate-gated ion channels, excessive activation of N-methyl-D-aspartate receptor (NMDA-R) leads to increasing intracellular calcium concentrations and the consequent production of damaging free radicals and activation of proteolytic processes that contribute to cell injury or death (136, 138). Xe has been identified to competitively inhibit the glycine site of NMDA-R, thus contributing to neuroprotective effects (128, 133, 139, 140) and it has carried out several clinical trials on brain–heart injury after cardiac arrest and achieved the positive results (141–143).

#### Xenon in Cardioprotection

Xenon is a new type of gaseous anesthetic with minimal hemodynamic side effects, thus, it is an ideal anesthetic for patients with heart damage (80, 144), while it has been suggested that Xe should be used with caution in patients with known intracranial hypertension (145–148). Global administration of 50 or 70% Xe only significantly reduced left ventricular systolic pressure and the maximum rate of pressure increase (dP/dtmax), the regional myocardial function and coronary blood flow in left anterior descending coronary artery- and left circumflex coronary artery-dependent myocardium were not changed; regional administration of 50 or 70% Xe only to the left anterior descending-perfused myocardium had no influence in global hemodynamics, regional myocardial function, and coronary blood flow in the circumflex coronary artery-dependent myocardium, while 70% xenon, rather than 50% xenon, reduced systolic wall thickening by 7.2 ± 4.0% and mean velocity of systolic wall thickening by 8.2 ± 4.0% in the left anterior descending coronary artery-perfused area, resulting in a small but consistent negative inotropic effect on beagle dog heart *in vivo* (149). Forty or 80% Xe did not significantly alter NO-dependent flow response, the electrical, mechanical, or metabolic effects in isolated guinea pig hearts, possibly due to no alteration of major cation currents in cardiomyocytes by Xe (150). Moreover, breathing 70% Xe had only minimal negative inotropic effects on rabbits with left ventricular dysfunction after coronary artery ligation (151). Schroth et al. also showed that 65% Xe did not alter myocardial contractility and the response to inotropic stimuli such as calcium, isoproterenol, or increase in pacing frequency in isolated guinea pig ventricular muscle bundles (144). The biological mechanisms of cardiovascular stability and unchanged muscle sympathetic activity during Xe anesthesia have been revealed by the *Peter Kienbaum* group; they found that the increased concentrations of norepinephrine at the synaptic cleft and in plasma by Xe in an NMDA-R-dependent mechanism contributed to the hemodynamic stability of patients during Xe anesthesia (152).

However, Xe (0, 20, 50, and 65%), in addition to basic intravenous anesthesia, has been shown to elicit downregulation of heart rate and cardiac output with no change in mean arterial pressure, decrease portal venous blood flow with no change in hepatic arterial blood flow, and reduce total hepatic oxygen delivery and venous hepatic oxygen saturation, but did not impair intestinal oxygenation in pigs (153, 154). 73–78% Xe with additional supplementation of pentobarbital and buprenorphine increased oxygen contents of hepatic venous blood in pigs (155). These indicate that the basic intravenous anesthesia might influence the effects of Xe on cardiovascular activities and hepatic oxygen contents.

Under pathological conditions, 70% Xe inhalation in the early stage of reperfusion can reduce infarct size after myocardial ischemia in rabbits (81); combined application of 20% Xe and 34°C hypothermia in early reperfusion can also reduce myocardial infarction size in rats (82) (Table 1). The mechanisms of Xe in cardioprotection have been relatively clear. Xe first activates mitochondrial KATP channel and phosphatidylinositol-dependent kinase-1 (PDK-1); these two activates PKC-ε, PKC-ε activates p38 MAPK, subsequently, two downstream targets of p38 MAPK, MAPK-activated protein kinase-2 (MAPKAPK-2/MK-2) and HSP27, are phosphorylated, and then, induces the translocation of HSP27 to particulate fraction and increases F-actin polymerization (80, 83, 84). Besides p38 MAPK, ERK1/2, and COX-2 are essential mediators of Xe preconditioning (85, 86); Xe can also induce the phosphorylation of Akt and GSK-3β, inhibit Ca^2+^-induced opening of mPTP, and preserve mitochondrial function (87). Similar to Ar, Xe also acts as an inhibitor of NF-κB activation and prevents adhesion molecule expression in TNF-α-treated ECs *in vitro* (156). The saturation point of Xe in water without a cage vehicle for encapsulation of xenon was 0.22 mM; when the cage molecule 2-hydroxypropyl-β-cyclodextrin (HPCD) was added, Xe solubility increased from 0.22 to 0.67 mM; supplement of this Xe-enriched solutions by gavage improved hypertension and left ventricular hypertrophy and dysfunction in aged apolipoprotein E (ApoE)-knockout mice fed high-fat diet (HFD) for 6 weeks (157).

#### Xenon in Renoprotection

Both the Ar and Xe have been shown as renoprotectants in kidney transplantation (158, 159). In addition, 70% Xe has been reported to improve kidney function and renal histology and decrease neutrophil chemoattractants expression in kidney, thereby attenuating the glomerular neutrophil infiltration in an accelerated and severe lupus nephritis model in female NZB/W F1 mice (160). This protective effects of Xe on kidney was mediated by enhancing renal hypoxia inducible factor 1-α expression; decreasing serum levels of antidouble-stranded DNA autoantibody; and inhibiting ROS production, glomerular deposition of IgG and C3 and apoptosis, nucleotide-binding oligomerization domain (NOD)-like receptor family protein 3 (NLRP3) inflammasome and NF-κB activation, and intercellular cell adhesion molecule-1 (CD54 or ICAM-1) expression in kidney (160). The role of Xe and other noble gases on the activation or inhibition of other forms of inflammasomes still need further investigation.

### Neon and Kr

The biological effects of Ne and Kr have been relatively few investigated in the past. Similar to He and Ar, Ne has also been shown to reduce the infarct area in rabbit model of myocardial I/R injury (73) (Table 1). Kr gas can promote the survival rate of Japanese quails embryos under acute hypoxia, Kr partial pressure of 5–5.5 kg/cm^2^ produces the narcotic effect on adult Japanese quails (161). However, in hypoxia/glucose deficiency injury model and in focal mechanical injury model of mouse hippocampal slices, only Ar and Xe showed the neuroprotective effects, while He, Ne, and Kr did not show neuroprotective effects (128, 133). Thus, the biological effects and mechanisms of Ne and Kr are worthy of further exploration.

### Radioactive Rn

Radon is an imperceptible natural occurring radioactive noble gas that exists in soil, water, and outdoor and indoor air; exposure to Rn accounts for more than 50% of the annual effective dose of natural radioactivity, it contributes as the largest single fraction to radiation exposure from natural sources (162, 163). Rn is a recognized pathogenic factor of human lung cancer, it is the second leading cause of lung cancer death after tobacco smoke (162). However, a certain dose of Rn has been reported for treating chronic musculoskeletal diseases, e.g., ankylosing spondylitis, osteoarthritis, or rheumatoid arthritis, these effects may be related to the regulation of oxidative stress and inflammation (163).

## Perspective

The noble gases are chemically inert because their outer electron orbitals are completely filled; however, they have been found to be very biologically active (159, 164). The noble gas family has emerged as the essential cellular or organic protectants such as in ECs, heart, brain, liver, kidney, and intestine; therefore, it protects against CCVDs (Figure 1).

Helium, Ar, and Xe displayed the neuroprotective effects on acute brain I/R injury models *in vivo* or *in vitro*. He, Ne, Ar, and Xe can reduce infarct size; Ar can improve the impaired left ventricular function in myocardial I/R injury animal models; however, the roles of other noble gases on left ventricular function under I/R or other pathological conditions still need further investigation (Table 1). It has been reported that oral administration of 6 weeks of Xe-enriched solution can be a promising nutraceutical strategy for cardiovascular protection (157). However, the effects of noble gases on chronic CCVDs and the side effects of long-time supplement of noble gases still need further investigation.

Besides competitively inhibiting NMDA-R by Xe in nervous system, modulation of cell death (mainly apoptosis), inflammatory or immune signals, oxidative damage, and gases interaction are the essential mechanisms of noble gases (Figure 1). The detail roles of noble gases on redox signaling, necrosis, autophagy, pyroptosis, and ferroptosis, which all play essential roles in CCVDs (165–167), and on other novel cell death types, such as alkaliptosis (168) and oxeiptosis (169), still need further investigation. The modulation of TLR4 signaling by He (89), NLRP3 inflammasome by Xe (160), and TLR2/4-mediated signaling by Ar (111) indicated that noble gases might act as essential modulators of innate immune signaling. Innate immune signaling is a complex cascade that quickly recognizes pathogen-associated molecular patterns (PAMPs) or damage-associated molecular patterns (DAMPs) through multiple germline-encoded cell surface or cytoplasmic pattern recognition receptors (PRRs), then, transmits signals through adaptors, kinases, and transcription factors, resulting in the production of cytokines (170–174). The mammalian host innate defense system utilizes more than 50 PRRs, which can be divided into two classes: the membrane-bound PRRs [including TLRs, C-type lectin receptors (CLRs), and receptors for advanced glycation end-products (RAGE)] and the cytosolic PRRs [including RIG-I-like receptors (RLRs), NOD-like receptors (NLRs), absent in melanoma 2 (AIM2)-like receptors (ALRs), and other nucleic acid-sensing receptors] (173, 174). Gasoimmunology, which investigates the effects of medical gases (such as NO, CO, H_2_S, SO_2_, H_2_, CH_4_, and noble gases) on innate immune signaling or on immune cells under both the homeostatic and perturbed conditions, will help us to open a novel door for medical gases investigation. Moreover, NO, CO, H_2_S, SO_2_, H_2_, and CH_4_ are essential endogenous gas molecules in modulating cardiocerebrovascular homeostasis (19–32). The cardioprotection of He is partially mediated by inducing NO production through eNOS in rabbits (78). It is not clear whether other noble gases can influence the levels and/or activities of these endogenous gases, if they can, what will happen to cardiocerebrovascular homeostasis and CCVDs?

It is not known the action forms of noble gases *in vivo*, by gases directly (in the alveolus where a gas phase exists) or dissolved non-electrolytes at very low concentration and with extremely weak interactions with other atoms/molecules. Therefore, as that of the small molecule signaling agents NO, CO, H_2_S, and their derived species, the physical or chemical interactions between noble elements and biological targets will be an important factor in their roles as signaling agents; thus, a fundamental understanding of the physics, chemistry, and biochemistry of noble gas atoms will be essential to understand their biological, physiological, or pathophysiological utility (175).

## Author Contributions

YZ: conceptualization. JZ and YZ: writing-original draft preparation. YZ, HY, WL, and JX: writing-review and editing. MB and YZ: visualization. YZ and HY: supervision. All authors have read and agreed to the published version of the manuscript.

## Funding

This study was funded by the National Natural Science Foundation of China (Grant nos. 81900376 and 81673772), the Natural Science Foundation of Guangdong Province (Grant nos. 2017A030313738 and 2018A030313657), Project of administration of Traditional Chinese Medicine of Guangdong province (20221116), the key discipline construction project for traditional Chinese Medicine in Guangdong province, and the construction project of inheritance studio of national famous and old traditional Chinese Medicine experts in 2021.

## Conflict of Interest

The authors declare that the research was conducted in the absence of any commercial or financial relationships that could be construed as a potential conflict of interest.

## Publisher's Note

All claims expressed in this article are solely those of the authors and do not necessarily represent those of their affiliated organizations, or those of the publisher, the editors and the reviewers. Any product that may be evaluated in this article, or claim that may be made by its manufacturer, is not guaranteed or endorsed by the publisher.
